# Extracellular Matrix as a Regulator of Epidermal Stem Cell Fate

**DOI:** 10.3390/ijms19041003

**Published:** 2018-03-27

**Authors:** Elina Chermnykh, Ekaterina Kalabusheva, Ekaterina Vorotelyak

**Affiliations:** 1 Koltzov Institute of Developmental Biology Russian Academy of Sciences, Moscow 119334, Russia; kalabusheva.e@gmail.com (E.K.); vorotelyak@yandex.ru (E.V.); 2 Department of Regenerative Medicine, Institute of Translational Medicine, Pirogov Russian National Research Medical University, Moscow 117997, Russia; 3 Faculty of Biology, Lomonosov Moscow State University, Moscow 119991, Russia

**Keywords:** extracellular matrix, epidermal stem cells, epidermal stem cell niche, keratinocytes, hair follicle, bulge

## Abstract

Epidermal stem cells reside within the specific anatomic location, called niche, which is a microenvironment that interacts with stem cells to regulate their fate. Regulation of many important processes, including maintenance of stem cell quiescence, self-renewal, and homeostasis, as well as the regulation of division and differentiation, are common functions of the stem cell niche. As it was shown in multiple studies, extracellular matrix (ECM) contributes a lot to stem cell niches in various tissues, including that of skin. In epidermis, ECM is represented, primarily, by a highly specialized ECM structure, basement membrane (BM), which separates the epidermal and dermal compartments. Epidermal stem cells contact with BM, but when they lose the contact and migrate to the overlying layers, they undergo terminal differentiation. When considering all of these factors, ECM is of fundamental importance in regulating epidermal stem cells maintenance, proper mobilization, and differentiation. Here, we summarize the remarkable progress that has recently been made in the research of ECM role in regulating epidermal stem cell fate, paying special attention to the hair follicle stem cell niche. We show that the destruction of ECM components impairs epidermal stem cell morphogenesis and homeostasis. A deep understanding of ECM molecular structure as well as the development of in vitro system for stem cell maintaining by ECM proteins may bring us to developing new approaches for regenerative medicine.

## 1. Introduction

Skin extracellular matrix (ECM) is composed of basement membrane (BM), which is a sheet-like structure separating dermis and epidermis, along with extracellular microenvironment of dermal fibroblasts and epidermal keratinocytes. ECM composition varies depending on the site in the skin. However its functions remains the same including cell adhesion and support, intercellular communication, regulation of cell differentiation, and all of the processes related to normal (homeostasis and aging) and pathological (wound healing, metaplasia, or malignancy) conditions [1]. The functional significance of ECM in controlling of epidermal stem cell fate has been highlighted in many studies [2,3,4]. Adult epidermal stem cells reside in specific stem cell niches, which play essential functions in regulating stem cell proliferation in order to maintain the epidermis homeostasis, and in protecting stem cells from depletion and undesirable stimuli [5]. Cell-cell and cell-ECM communication within the niche maintains stem cells in undifferentiated state or promote their differentiation. At least three epidermal stem cell niches have been found in the skin: the basal layer of interfollicular epidermis (IFE), hair follicle (HF) bulge, and the base of the sebaceous gland [6,7,8,9]. Little is known about the niche in the IFE. Stem cells are located among the cells of the basal layer and are in contact with the BM. Depending on body site stem cells of human glabrous, epidermis can be located at the base of rete ridges [10,11] or overlying the tip of dermal papilla [12,13]. HF stem cells reside in special area of upper HF called bulge. In steady state, IFE is not replenished from the HF bulge, but epidermal wounding causes upward migration of bulge progeny to the wound [14,15]. Conversely, interfollicular stem cells are also exhibit multipotent properties and can regenerate HFs upon wounding [14]. The mechanism underlying the maintenance of sebaceous stem cells is not well understood. Renewal of the gland may occur by unipotent progenitor cells located at the periphery of the sebaceous gland or by HF stem cell progeny activated and mobilized to regenerate the sebaceous gland [16,17]. The common feature of epithelial stem cells from all locations is residing within the basal layer of epidermis closely contacting with BM rich in ECM proteins and growth factors [18]. Epidermis is self-renewed by the division of basal keratinocytes with subsequent multiplication in transit amplifying compartment and terminal differentiation in the superficial layers. To accomplish cornification, dividing basal cells have to detach from BM, move outward, go through multistep process of differentiation, and finally, die [19]. The molecular and cellular signals orchestrating specific cell-fate decisions may involve the ECM, intrinsic cellular signaling pathways as well as the regulation by hormones and surrounding stromal cells [17]. Stem cell compartment is maintained by asymmetric divisions in basal layers, which are ensured by the proper orientation of the mitotic spindle which should be perpendicularly to the BM [20,21,22,23], generating one cell for the basal layer and another one for the suprabasal position. Keratinocytes in the basal layer closely contact with the BM and the associated growth factors through integrins and receptors, while suprabasal progeny loses this contact being removed from the stem cell niche and acquires differentiation fate [24]. BM components and cell surface transmembrane integrins retain stem cells in the niche as well as control cell polarity, anchorage, proliferation, differentiation, and migration [25]. Notably, the absence of β1-integrin or α-catenin results in the randomization of spindle alignment and misoriented cell divisions, underlying the requirement of the BM and cell-cell junctions in this process [23].

Here, we make an attempt to review the latest advances in the field of epidermal stem cell biology, focusing on the extracellular environment components that may influence stem cell fate. We highlight that the destruction of ECM components impairs epidermal stem cell morphogenesis and homeostasis. We cover the possible ways of in vitro studying ECM influence on epidermal stem cells behavior. Understanding the signals in the niche that regulate stem cell behavior is important for applications, such as tissue engineering and also for elucidating mechanisms of undesirable effects, such as tumor unregulated proliferation.

## 2. Skin Extracellular Matrix (ECM) Components

The BM makes a major contribution to the fate of stem cells via direct contacts with them. BM components are synthesized by collaborative efforts of basal keratinocytes and dermal fibroblasts [26,27]. The main components of epidermal BM are fibrous forming proteins (type IV and VII collagens, elastin), glycoproteins (laminin, fibronectin), nidogen, and proteoglycans (heparin sulfate, perlecan) [28]. Fibrous proteins form fibrils from protein monomers and provide tensile strength, glycoproteins mediate cell-cell and cell-matrix interactions, proteoglycans withstand compressional forces. Keratinocytes are associated with BM by special linker proteins—integrins, which are the main receptor proteins that cells use to both bind to and respond to the ECM. The molecules of the ECM can bind to growth factors and their receptors and mediate signaling. For example, fibronectin binds fibroblast growth factor (FGF), vascular endothelial growth factor (VEGF), and platelet-derived growth factor (PDGF) [29]. Epidermal growth factor receptor (EGFR) interacts with tenascin-C and laminin 5, promoting cell proliferation. In contrast, EGFR activation by proteoglycan decorin inhibits mitogenic signaling in fibroblasts and endothelial cells [30]. Growth factors together with proteases regulate ECM structure: growth factors initiate deposition of ECM components, while proteases enable their degradation, ensuring the remodeling of ECM that is required for cell movement [31].

In dermis, fibril-forming collagens (type I and III) are the main elements of ECM. They are associated with other ECM proteins and proteoglycans to form fibrilar network.

Laminins are essential for the initial assembly of the BM in vivo [32]. Laminin-511 (laminin 10) and laminin-332 (laminin 5) are the most common laminins of developing and adult skin. Both are secreted by keratinocytes and are accumulated in the region of the BM surrounding the HF, including a bulge region [3]. Laminin-511 is crucial for HF development [33], but we know much less about its role in interfollicular development and homeostasis [34]. Laminin-332 and laminin-511 can bind α3β1, α6β1, and α6β4-integrins [35,36,37], however, the association of laminin with α6β4-integrin is most critical for the stability of keratinocyte contact with the BM. Mice with a deletion of α6 and β4-integrin subunits die shortly after birth, they have numerous blisters on the skin, due to the lack of hemidesmosoms. Inactivation of laminin gene resulted in multiple embryonic defects [28,38]. Mutations in any of the three laminin subunits can cause blistering with a split through the BM itself [39]. Laminin 511 is important for HF morphogenesis while knockout of laminin 332 chains in mice causes neonatal death with severe skin blistering resembling lesions of patients with junctional epidermolysis bullosa caused by mutations in the same gene [40].

Fibronectin is involved in a number of cellular mechanisms important to cell adhesion, growth, migration, differentiation, wound healing, and matrix assembly, and serves as a binding site for a number of ECM components. In the skin, fibronectin can function as a general cell adhesion molecule by binding cells to collagen or proteoglycan. It inhibits the terminal differentiation of human keratinocytes [41]. Inactivation of the fibronectin gene results in the early embryonic lethality of mice [42], while altered fibronectin expression, degradation, and organization has been associated with a number of diseases [43,44].

Type IV collagen is an essential component of the BM of skin and HFs. Its role in maintaining epidermal stem cells has been suggested in many studies. Human putative epidermal stem cells could be enriched on the basis of their rapid adherence to type IV collagen [45]. Inhibition of miR135b, an effector of type IV collagen, can increase the proliferative potential of human basal keratinocytes and improve the epidermal stem cell [46]. It was suggested that the presence of type IV collagen in BM is important for stem cell maintenance in skin [47]. Defects in the type IV collagen are not compatible with early embryonic development. Type IV collagen primarily provides mechanical support [32].

Heparan sulphate, which is the main polysaccharide component of the ECM, is mainly distributed in the BM zone of both skin and HF, with a decreased level at the hair bulb periphery, as well as in dermal papilla [48]. Heparan sulfate polysaccharide chains can bind to a large number of bio-molecules that are involved in HF biology. Heparanase, which is an endoglycosidase that cleaves heparan sulfate, is expressed mainly in the outer root sheath (ORS) areas along the hypothesized migration route of the transit-amplifying cells, and may regulate hair growth promoting cell detachment and the release of heparin bound growth factor [49]. Heparanase expression depends on HF cycle stage with increased expression in anagen and decreased expression in catagen [49]. Overexpression of heparanase was shown to improve hair growth in mice skin [48]. The absence of heparanase in HFs led to altered differentiation of the cells during anagen, which could hinder regression into catagen [50]. Thus, heparanase could be crucial for anagen maintenance and human HF homeostasis [48,50].

Hyaluronan is localized in the dermis in large quantities, but it also found in relative high concentrations in the extracellular spaces of basal, spinous, and granular layers of epidermis with proliferating basal regions containing the majority of hyaluronan. The terminally differentiated cells of stratum corneum usually lack hyaluronan. This localization suggests a role of hyaluronic acid in keratinocyte proliferation [51]. Hyaluronan increases in the mouse epidermis when keratinocytes start to stratify on day E15, remains high until birth, and then rapidly declines. In epidermis, hyaluronan content is rapidly increased after tissue wounding [51]. All of these factors may indicate that hyaluronan influences epidermal stem/progenitor cells in tissue development.

Integrins are the major receptors for ECM proteins, their interaction with growth factor and cytokine receptors is an important signaling mechanism [52]. Being firstly recognized as physical linkers to BM and ECM, integrins, like ECM molecules themselves, play their particular roles in the regulation of tissue homeostasis in epidermis. Integrins differ in structure and functionality. The α6β4 integrin was found to be a central player in IFE anchorage to BM, while β1-containing integrins are necessary for HF and stem cell maintenance [2,53,54]. At the same time, one could suggest that certain integrin functions have some complementary signaling pathways. At least, stem cell maintenance is not solely provided by β1-integrin signaling as its activation is not sufficient to increase stem cell compartment in vivo [55]. Multiple mice with knockouts in integrin genes help us to elucidate their role in keratinocyte adhesion to BM. Some deletions have striking effects, as in case of α6 and β4 subunits when mice with deletions in these genes die shortly after birth suffering of skin blistering and flat stratified epithelia without hemidesmosomes. Humans with analogous mutations develop epidermolysis bullosa [56].

The interaction of α4β1 and α9β1 integrins with ECM glycoprotein EMILIN1 was shown to be important for maintaining a correct skin homeostasis [57].

Proteases can remodel the ECM architecture inducing the local release of soluble growth factors from their insoluble anchorage. The degradation of the ECM is key for tissue growth and development [58]. Many proteolytic enzymes, such as serine proteases, are able to lyse individual components of the ECM, but only matrix metalloproteinases (MMPs) can destroy almost all of the ECM structures, including collagens, fibronectin, and laminins [59], thus providing their turnover as well as regulating lifespan and activity of cell-surface receptors, including integrins and ECM-immobilized growth factors and cytokines. It was shown that the inhibition of the type IV collagenases, MMP-2 and MMP-9, retarded hair regrowth after depilation, and the authors demonstrated that this was accompanied by a decrease in expression of VEGF, IGF-1, and TGF-β [60]. The activity of MMPs is regulated by their specific inhibitors, known as tissue inhibitors of metalloproteinases (TIMPs). MMP-2 and MMP-9 activities are specifically inhibited by TIMP-2 and TIMP-1, respectively, which may be vital in the regulation of HF. Indeed, the fluctuation of MMP-2 and MMP-9 expression levels was shown to occur throughout the hair growth cycle [61]. MMP-2 and TIMP-2 were found in all of the structures of the HF, while MMP-9 and TIMP-1 are restricted to certain areas of the HF, such as the sebaceous gland and the inner root sheath [61]. These results suggested that MMP-2 and MMP-9 might play an important role in the hair growth cycle. The expression of serine protease kallikreins (KLKs) 5, 7, and 14 is characteristic of human scalp telogen club hairs, and it has been suggested that KLKs play a role in releasing the club hair from the follicle [62].

## 3. ECM Components as Epidermal Stem Cell Markers

As ECM components of skin are involved in regulation, and the maintenance of epidermal stem cells it is not surprising that the latter may be identified by expression of certain ECM receptors. Integrins are among such markers [63]. They are transmembrane glycoproteins that primarily mediate the attachment of basal keratinocytes to ECM proteins found in the BM. In vivo, basal keratinocytes express the β1-integrins α2β1 (receptor for collagen and laminin), α3β1 (receptor for laminins), α5β1 (the fibronectin receptor), as well as the integrin α6β4 (receptor for laminins) [64]. Expression of the β1-integrin subunit was proposed as one of epidermal stem cell markers. Keratinocytes that form holoclones with the highest efficiency are mostly brightly stained for β1-integrin [63]. HF bulge also contains cells with high β1-integrin expression [13,65,66]. A high level of α6-integrin expression indicates a high capacity for cell proliferation [67]. Stem cells adhere rapidly to type IV collagen, fibronectin, and the ECM that is deposited by cultured keratinocytes [13]. Different techniques based on adhesion characteristics, have been proposed to isolate epidermal stem cells [68]. High expression level of β1-integrin makes stem cells able to adhere quicker than the others to type IV collagen [13]. Selected by this method keratinocytes were highly proliferative and were able to reconstruct a fully differentiated epidermis when grafted onto nude mice, suggesting that this fraction of the basal layer contains epidermal stem cells [13].

Some techniques use several basal cell markers simultaneously to enrich stem cell population in basal cells. Basal keratinocytes with high growth potential and the capacity for generating a stratified epidermis can be isolated based on their high adhesion characteristics on a type I collagen-coated substrate and high cell-surface expression of α6-integrin [69]. Negative selection may be also useful. The expression of desmosome protein desmoglein 3 (Dsg3) can be used as a negative marker for keratinocytes with high clonogenecity and proliferative potential. Double sorting to select β1bri/Dsg3dim allowed for authors to obtain highly enriched population of putative epidermal stem cells [70]. There are other variants of double selection with the use of negative markers. Cells with the phenotype α6briCD71dim display a quiescent state, the greatest regenerative capacity and represent a minor subpopulation (≈10%) of immature epidermal cells [71]. α6briCD71dim phenotype showed a better enrichment of epidermal stem cells based on colony forming efficiency test, when compared to cells isolated by a rapid adherent method [72]. 

Melanoma chondroitin sulphate proteoglycan (MCSP) has been proposed as a putative stem cell marker due to it heterogeneous expression in the basal layer of human IFE and in the ORS of HFs [73,74]. One putative function of MCSP is its contribution to stem cell clustering by promoting cell-cell adhesion [73].

A significant problem in these types of studies is the lack of reliable epidermal stem cell markers, making adhesion-based approaches elusive, not allowing for one to distinguish stem cells from their transit-amplifying progenies. Knowledge of these markers may lead to rapid progress in this area.

## 4. ECM Role in Epidermal Stem Cell Niches

While many ECM components are similar across different tissues, even within specific regions of the skin and at different developmental stages, there is considerable anatomical and molecular variation in their expression [75]. For example, laminins and integrins show heterogeneity in different regions of the skin BM [76,77]. Such variation in BM components and integrin expression provides many distinct cell adhesive properties and may potentially create an environment that is appropriate for stem cell maintenance. Laser ablation studies showed the importance of niche microenvironment, illustrating that when one cell is removed from its niche, another nearby cell can replace it, even if it comes from a more committed progenitor population [78,79]. Acellular materials with ECM may regulate the proper fate and differentiation of stem cells in the context of tissue engineering [80]. Decellularized tissue with the remaining tissue and site-specific ECM scaffold can induce stem cells to differentiate into cell types belonging to the tissue source of ECM [81].

Changes in ECM components intensively occur during embryogenesis, in response to the infectious agents, as well as with various pathological conditions. This process in the ECM is mediated by integrins as well as syndecans—proteoglycans of ECM or through protein-enzymes with the ability to modify ECM enzymes—MMPs [76]. Altered or aged niches reduce ability to maintain stem cell properties [82]. This decline in stem cell function may be linked to changes in post translational modification of ECM proteins. A recent study showed increased carbamylation and glycation of type I collagen and elastin in aging skin, suggesting that these post translational modifications are associated with aging [83]. Modifying the aged microenvironment with the young niche components can reverse the proliferation and renewal of the stem cells to the “younger” level [84].

Signaling pathways involved in ECM-epidermal stem cell interactions are not sufficiently studied to date. Attention of scientists is mainly focused on signaling cascades, known to be crucial for epidermal stem cell homeostasis and skin morphogenesis, including Wnt, TGF-β/BMP, Notch, Hedgehog, and FGF.

Wnt/β-catenin signaling is well recognized to play a role in controlling epidermal stem cell maintenance and fate decision. It participates in multiple processes in the skin, including cell-matrix interactions within the niche and outside.

One of the ECM proteins in IFE that is linked to Wnt signaling is the component of hemidesmosomes, and, in part, non-hemidesmosomal type XVII collagen, the target protein in junctional epidermolysis bullosa. It balances proliferation of IFE in neonatal paw mouse skin through the activation of Wnt pathway [85]. Col17a1^−/−^ P1 mouse epidermis demonstrated a decreased expression of genes that are related to Wnt activation, whereas the expression of the inhibitory Wnt4 was elevated [85], indicating collagen XVII interaction with this pathway. However, exact mechanisms of interconnection with Wnt signaling remain unclear. Although epidermal hyperproliferation, which accompanied the loss of COL17, was transient it is rather difficult to explain this effect, as it was shown that Wnt activation, not loss, stimulated epidermal proliferation and stem cell maintenance in autocrine manner [86]. On the other hand, one should keep in mind that a wide range of Wnt activators and inhibitors are secreted in the skin participating in the complex interplay, including other pathways, ECM molecules, and cell-cell interactions.

Regulation of Wnt pathway activity in the IFE is complexly regulated by the epidermal BM, which produces controversial signals towards cell proliferation and morphogenesis. Epidermal activation of β-catenin causes the formation of ectopic HFs and remodeling of the dermis, which is accompanied by the preferential expression of collagen 11a1 [87]. Changes to ECM in this case are mediated by TGF-β2, and to a less extent by FGF2, secreted by activated epidermis [88].

ECM of dermal fibroblasts play essential role in BM genesis and thus may contribute to stem cell niche homeostasis by influencing local tissue architecture and signalling microenvironments. Fibroblasts deposit collagen and elastic fibres of the ECM in connective tissue, produce BM components and assist in BM assembly [26], exhibiting an essential role for epidermal growth [89]. A number of distinct differentiated mesenchymal cell types that have different origins, locations, and functions have been uncovered [90]. When considering their differences in ECM synthesis and growth factor and cytokine secretion, the papillary and reticular dermal fibroblast populations possess distinct abilities to promote keratinocyte growth, migration, and differentiation, as well as influence the ECM synthesis and remodeling in the dermis [91,92].

## 5. Hair Follicle (HF) Stem Cell Niche

The HF stem cell niche, the bulge region, is anatomically distinct and allows for the investigators to understand niche characteristics more precisely. Many studies have been focused on mouse HF stem cells transcriptomic signature that enriched genes are highly associated with ECM organization, mainly laminins, collagens, glycoproteins, ECM proteases, and protease inhibitors [93,94,95]. The gene expression of the following ECM component proteins was up-regulated between HF bulge stem cells and the differentiated epidermal cells: metallopeptidase, cingulin, type IV collagen α3 and α4 chains, fibulin-1, hyaluronan and proteoglycan link protein 2 (HAPLN2), integrin β8 [96], tenascin-C, α chains of type VI and type XVIII collagens, biglycan, syndecan 1, and others [94,95]. There are also the differences in the expression of ECM proteins between HF stem cells and other epidermal stem cells [96].

Several components of ECM act through Wnt pathway to regulate hair epidermal stem cell activity. For example, type VI collagen is highly expressed ECM component contributing to bulge stem cell niche; it affects hair cycle and possibly is involved in wound healing. Col6a1^−/−^ mice have delayed hair cycling and growth under normal conditions. On the contrary, wound-induced hair regrowth is increased in the absence of this type of collagen. It was demonstrated that purified type VI collagen protein rescued normal hair regrowth upon wounding by regulating the Wnt/β-catenin signaling pathway [4]. Glycoprotein tenascin-C increases the local concentration of Wnt3a near cell surface intensifying Wnt signaling and maintaining the stem fate of CD34-positive bulge cells [97]. It is a good example of linkage between ECM molecule and signaling pathway via immobilization of ligands on ECM. Tenascin-C knockout mice have no visible defects of the integumentary system, but detailed examination of whisker follicles showed an increased number of adipose and mast cells within the follicle and in the loose mesenchyme, respectively. The authors suggest that the absence of tenascin-C results in lower concentration of Wnt3a in proximity to cells, and, thus, in decreased level of Wnt signaling, which, in turn, does not prevent bulge stem cells from aberrant adipogenesis and mast cell differentiation as they originate from neural crest and have potential to these lineages of differentiation.

Laminin-511 (laminin-10) was found as a crucial signal in HF development, it is up-regulated in growing HF and the skin of Lama5^−/−^ mice failed to elongate hair germ that resulted in the complete HF regression. These defects could not be connected with cell detachment from the BM thus suggesting special role of laminin 511 in HF morphogenesis [98]. Importantly, the precise ratio between laminin-332 and laminin-511 is essential for the activation of stem cells within the bulge niche of HF and maintenance of stem cell homeostasis [3].

There is one more interesting ECM protein specific for HF stem cell niche—nephronectin, was originally found as a functional ligand of α8β1-integrin in kidney development [99]. Nephronectin is permanently expressed in the bulge BM by epidermal keratinocytes, whereas its receptor, α8β1 integrin is expressed in the HF dermal papilla. In the HF, nephronectin induces arrector pili muscle differentiation and anchorage to the bulge. Thus, the bulge ECM represents a “double” niche, both for HF stem cells and smooth muscle progenitors [100]. As it was shown by chromatin immunoprecipitation assays, nephronectin is the direct target of Tcf4, which is the bulge Wnt effector. Inhibition of Wnt/β-catenin signaling by interfering into formation of β-catenin/Lef/Tcf complexes led to decreased nephronectin deposition in the bulge and hair germ. In this case, the expression of nephronectin family member EGFL6/MAEG in the upper bulge is compensatory increased, and muscles insert in that region [101]. Interestingly, expression of nephronectin increased in IFE of K14ΔNLef1 mice, indicating an opposite output of Wnt/β-catenin signaling activation depending on the site of its action.

Type XVII collagen, which is a hemidesmosomal transmembrane collagen that is highly expressed by HF stem cells, is involved in the self-renewal of not only HF stem cells, but also melanocyte stem cells, maintaining their quiescence and immaturity [101]. TGF β signaling is involved in type XVII collagen-dependent melanocyte and bulge stem cells maintenance [101]. Col17a1-null mice have defect melanocyte stem cell niche where bulge epidermal stem cells-derived TGF β signaling is downregulated by eight weeks of age. This causes hair graying, thus demonstrating the important role of type XVII Collagen in the HF/melanocyte stem cell niche [101,102]. Moreover, HF cycle is dramatically affected by the absence of Col17a1—while the first telogen is normal, the second one is significantly shortened, followed by rather prolonged anagen phase. Stem cells population gradually disappears from nearly all follicles. Failure of stem cell compartment causes follicle atrophy and degradation. After analysis of bulge cell proliferation and differentiation with subsequent experiments on clonal growth in culture the authors conclude that Col17a1-null HF stem cells are unable to maintain quiescence and loose self-renewing ability. This effect may be related to some unknown regulatory mechanisms that are provided by type XVII collagen, whether by physical attachment of cells to the basal lamina or through unidentified signaling pathway. Notably, bulge keratinocytes in aged mice are characteristic of reduction or loss of type XVII collagen, implicating the type XVII collagen in age-related HF alterations [102].

Examination of the gene signature of mouse bulge stem cells revealed such tendon-related genes, as Scx (scleraxis), Mitf, Igfbp5, Fbln1 (fibulin-1), Postn (periostin), Tnc (tenascin-C), Sparc, Igfbp6, and Fgf18 [94,95]. Immunohistochemical analysis revealed periostin and fibulin-1 localize to the bulge rather than along the entire ORS [100]. Fibulin-1 expression was found in fibroblasts [103] and in the epithelial placode and the mesenchymal condensate in developing vibrissa follicles [104]. In telogen HF, the only bulge was positive for Tenascin-C, while in anagen, its expression was more widespread [100,105,106].

As it was mentioned above multiple signals are transduced through integrins and integrin-associated proteins with multidirectional effects on skin development and homeostasis [107]. Integrin-ECM interactions are extensively involved into stem cell fate regulation, demonstrating crucial dependence on adhesion and proper cell-ECM contact [76]. As an example, it was found that integrin activator Kindlin-1 suppresses Wnt signaling and its absence provokes elevated levels of Wnt ligands and receptors accompanied by expansion of bulge stem cell compartment and their elevated proliferation [108]. This effect was not related to its binding to β1-integrin. At the same time, Kindlin 1 stimulates TGF β release mediated by αvβ6-integrin, which is necessary for stem cell quiescence. 

Another protein binding integrins to actin cytoskeleton is an integrin-linked kinase (ILK). According to data obtained in mice, ILK provides proper balance between epidermal proliferation and differentiation, since its absence causes epidermal hyperproliferation [3].

The specific position that a stem cell occupies within the niche can influence its behaviour. The location of a stem cell within the HF niche can predict whether it is likely to remain uncommitted, generate precursors, or commit to a differentiated fate. Within the bulge, stem cells in the upper half of the bulge are favored to remain quiescent or to self-renew. In contrast, cells that are situated in the lower bulge are more likely to proliferate and generate ORS lineages [109].

## 6. Study of ECM Role In Vitro

There are only a few stem cells in each tissue; skin stem cells represented less than 1% of keratinocytes [110]. In vitro stem cells abandon the niche, and then begin to lose their stem cell characteristics; the size, proliferation, and migration of cells are increased [111]. However, several studies have demonstrated proper culture systems that provided the survival of epidermal stem cells [112,113]. With the great potential of stem cells in regenerative medicine, finding ways to maintain and expand the quantities of stem cells by in vitro culture is of particular interest. Moreover, in vitro models allow overcoming the drawbacks connected to in vivo compensatory mechanisms. Indeed, the lack of one specific ECM component in vivo may not directly affect stem cell behavior because it can be masked by the activation of another ECM component. For example, the loss of α2β1-integrin expression is compensated by an increase in the expression of other integrins with similar properties [114]. Similarly, β1-integrin can compensate the absence of dystroglycan, and vice versa [115]. Traditional approaches to investigate cell-ECM interactions are represented by two-dimensional (2D) keratinocyte culture on surfaces coated with adhesive proteins or a mix of proteins. Certain ECM molecules have been found to play essential role in maintaining cells in undifferentiated state. Stem cells express high levels of α2β1, α3β1, α5β1-integrins, and adhere rapidly to type IV collagen, fibronectin, and the ECM that is deposited by cultured keratinocytes [68]. In culture, β1-integrin activation suppresses the terminal differentiation of keratinocytes [116]. Laminin-511 can support embryonic stem cell self-renewal in the absence of differentiation inhibitors and at low cell density [117,118]. Engineered 2D micropatterned substrates as well as hydrogels helped to elucidate the behaviour of epidermal cells [119,120]. It was shown that the matrix area [119] and its stiffness [120], rather than ECM concentration or composition are essential for keratinocyte differentiation, suggesting that biophysical factors may be more important in determining the stem cell fate. To this end, it may be of interest that changes in ECM mechanics and in cell shape are transmitted to the cell nucleus and regulate gene transcription programs [121].

A drawback of 2D cell culture is that it does not adequately reproduce natural three-dimensional (3D) environment of cells and does not provide the complex evaluation of growth factors and molecules associated with BM. Moreover, spatial organization of the cell surface receptors engaged in interactions with surrounding cells, as well as stiffness characteristics of niche environment, which may affect gene expression and cellular behavior, can also not be addressed. Over the last years, a variety of 3D cell culture systems in stem cell biology has been developed. Such 3D models provide excellent in vitro tools to study cellular responses replicating in vivo environment [122]. 3D cultivation system demonstrated the maintenance of limbal epithelial stem cells [123] and gastric stem or progenitor cells within the stem cell niche over the long term without addition of special reagents [124]. To replicate the basic skin system, organotypic cocultures were developed based on type I collagen matrix inhabited by fibroblasts and keratinocytes. In this 3D coculture system, a normal epidermal phenotype and BM membrane structure could be reconstructed [125,126]. Using of natural ECM proteins as scaffold resemble the native in vivo environment cells are exposed in, bioengineered synthetic scaffolds may exhibit any required mechanical and physical characteristics mimicking the in vivo stem cell niche [127].

Looking forward, development of scaffolds containing variety of cell types specific for dermis, exhibiting the required mechanical characteristics may resemble the in vivo situation more closely, providing a valuable asset to stem cell biology and regenerative medicine. New surface-proteomic screening approaches examining interactions between cells and different biomaterials may also be useful for the investigation of in vivo properties of epidermal cells [128].

A growing number of studies indicate the extremely important role of microenvironment in the functioning of stem cell niche. An understanding of the regulatory mechanisms and functioning of stem cell niche is not only of fundamental interest, but also of importance for therapeutic applications, as it could help to develop new applications in regenerative medicine. ECM influences the behavior of stem cells during the initiation and progression of degenerative and oncological diseases. Thus, ECM biology and niche factors should be considered when targeting the niche for the therapy of these diseases.

An emerging focus of regenerative medicine is on the development of synthetic biomaterials that can be used as a three-dimensional cell microenvironment reconstructing the regulatory characteristics of the natural ECM. Unfortunately, most of the materials that are currently used to create the matrix do not reflect the complexity of the ECM and therefore are unable to restore the morphology and function of the transplanted cells. The collaboration between materials science and cell biology methods will eventually lead to the creation of such structures from cells and matrix materials, recapitulating natural microenvironment that supports the viability and function of cells.

## Abbreviations

ECM	Extracellular Matrix
BM	Basement Membrane
IFE	Interfollicular Epidermis
HF	Hair Follicle
MMPs	Matrix Metalloproteinases
ORS	Outer Root Sheath
Dsg3	Desmoglein 3
MCSP	Melanoma Chondroitin Sulphate Proteoglycan
ILK	Integrin-Linked Kinase
FGF	Fibroblast Growth Factor
VEGF	Vascular Endothelial Growth Factor
PDGF	Platelet-derived Growth Factor
EGFR	Epidermal Growth Factor Receptor
